# Investigation of Effective Parameters on Size of Paclitaxel Loaded PLGA Nanoparticles

**DOI:** 10.15171/apb.2018.010

**Published:** 2018-03-18

**Authors:** Fatemeh Madani, Seyedeh Sara Esnaashari, Basil Mujokoro, Farid Dorkoosh, Masood Khosravani, Mahdi Adabi

**Affiliations:** ^1^Department of Medical Nanotechnology, School of Advanced Technologies in Medicine, Tehran University of Medical Sciences, Tehran, Iran.; ^2^Student's Scientific Research Center, Tehran University of Medical Sciences, Tehran, Iran.; ^3^Department of Medical Nanotechnology, School of Advanced Technologies in Medicine, International Campus, Tehran University of Medical Sciences, Tehran, Iran.; ^4^Department of Pharmaceutics, Faculty of Pharmacy, Tehran University of Medical Sciences, Tehran, Iran.

**Keywords:** PLGA, Paclitaxel, Nanoparticle, Size

## Abstract

***Purpose:*** The size of polymeric nanoparticles is considered as an effective factor in cancer therapy due to enterance into tumor tissue via the EPR effect. The purpose of this work was to investigate the effective parameters on poly(lactic-co-glycolic acid)-paclitaxel (PLGA –PTX) nanoparticles size.

***Methods:*** We prepared PLGA-PTX nanoparticles via single emulsion and precipitation methods with variable paremeters including drug concentration, aqueous to organic phase volume ratio, polymer concentration, sonication time and PVA concentration.

***Results:*** PLGA-PTX nanoparticles were characterized by dynamic light scattering (DLS) and scanning electron microscopy (SEM). The results exhibited that the diameter of nanoparticles enhanced with increasing drug, polymer and PVA concentrations whereas organic to aqueous phase volume ratio and sonication time required to the optimization for a given size.

***Conclusion:*** The precipitation method provides smaller nanoparticles compared to emulsion one. Variable parameters including drug concentration, aqueous to organic phase volume ratio, polymer concentration, sonication time and PVA concentration affect diameter of nanoparticles.

## Introduction

Cancer is a major global cause of morbidity and mortality which is estimated that the incidence of all new cancer cases will reach 22 million by 2030 in worldwide.^1^ Chemotherapy is a versatile cancer treatment modality due to its application as first line,^2,3^ adjuvant^4^ and/or palliative therapy^5^ in the fight against different cancers. In addition, chemotherapy is easier to administer and less invasive compared to other clinical cancer treatment modalities such as surgical removal and radiotherapy.‏ Unfortunately, since the‏ efficacy of most chemotherapeutic drugs‏ is dose dependent, severe‏ chemo-induced side events have been observed at higher doses.^6-8^ Thus targeted delivery of drugs with minimum non-specific exposure is essential for successful chemotherapy. Tumor targeting chemotherapy can be‏ accomplished by exploiting the diseases’ pathophysiology such as unique or overexpressed molecules^9^ and leaky tumor vasculature.^10^

Therapeutics can be passively targeted to the hyper-permeable tumor vasculature commonly observed on most cancers. Moreover, the absence of lymphatic drainage in tumors leads to retention of accumulated therapeutic agents within the tumor tissue.^11,12^ This unusual extravasation, accumulation and retention of expediently sized therapeutic molecules within tumor tissue is called enhanced permeability and retention (EPR) effect.^13^ In addition, nano-sized drug carriers can simultaneously deliver higher amounts of drugs with lower unspecific toxicity, without loss in therapeutic activity. Examples of these biocompatible nano-scale drug carriers include solid lipid nanoparticles (SLN),^14^ liposomes,^15^ micelles,^16^ nanobubbles^17^ and polymers.^18,19^ Among these, an extensively studied family of materials in the fabrication of biocompatible nanostructures is polymers.^20,21^ Polymeric nanostructures possesses several advantages such as simple synthesis techniques, ability to carry a wide payload of therapeutic agents and biodegradability.^22^ A wide range of synthetic and natural polymers have been investigated for a variety of biomedical applications such as tissue engineering,^23,24^ bioimaging^25^ biosensors^26-28^ and drug delivery.^29,30^ Synthetic polymers, notably,‏ PLGA and its co-monomer PLA are widely used in the synthesis of‏ nano-sized drug delivery systems. Flexible synthesis techniques enable the tailoring of nanoparticle properties such as size,^31^ drug loading^32^ and in-vivo drug release.^33^

Among a legion of anti-cancer drugs, paclitaxel is a potent chemo-agent used in the treatment of several solid tumors including ovarian cancer, breast cancer, AIDS related kaposi sarcoma and lung cancer. Further investigations on the efficacy of paclitaxel against gastrointestinal cancer,^34^ glioblastoma^35^ and pancreatic cancer^36^ have yielded promising results. However, a major clinical limitation of paclitaxel is the drugs’ poor solubility in water.‏ Therefore‏ biocompatible nano-sized colloidal structures offer‏ safer alternative paclitaxel delivery vehicles.^37-40^

Since most synthesis nanoparticle and drug loading techniques are well established, current scientific focus is increasingly being directed towards optimization of various parameters to obtain effective formulations. Therefore, for EPR targeting, it is of great interest to determine the various input parameters which affect the diameter of nanoparticles. Meanwhile, it is also important to be cognizant of the rate limiting size-dependent physiological processes which may affect the intra-tumoral accumulation of nanoparticles. Nanoparticle sizes less than 30 nm are prone to renal filtration^41^‏ whilst sizes larger than 250 nm are ideal candidates for phagocytosis.^42^ Therefore, the effective therapeutic window for EPR targeting may be considered between 50 and 200 nm. Thus the aim of this work was investigate the various parameters which affect the diameter of PTX loaded PLGA nanoparticles for effective EPR targeting. PLGA nanoparticles were chosen for this study due the simplicity and flexibility of synthesis techniques such as nanoprecipitation and emulsion/solvent evaporation.^43,44^

## Materials and Methods

Paclitaxel was purchased from sigma. PLGA (50:50, MW 30000 g mol^−1^) was bought from Shenzhen Esun Industrial Co., China. Dichloromethane (DCM) and acetone (99%) supplied by Carol Erba. Polyvinyl alcohol (PVA), fully hydrolized (MW 60000 g mol^−1^) was obtained from Merck (Germany). All solutions were prepared using deionized water.

### Preparation of paclitaxel-loaded nanoparticles by single emulsion

Nanoparticles were prepared by single emulsion (O/W) method. The PVA polymer was dissolved in deionized water as aqueous phase under continuous magnetic stirring at 40 °C for 5 h to obtain a homogenous solution. Different amounts of paclitaxel and PLGA were dissolved in DCM and stirred for 1 h at room temperature. Then the organic phase poured in PVA solution (30 ml) and stirred for 30 min at room temperature. Then emulsion was sonicated using probe sonication (Top Sonics Ltd., Co., Iran) for 4 minute. After evaporation of the organic solvent overnight, the nanoparticles were collected by centrifugation (Eppendorf centrifuge) at 12000 rpm for 20 min at room temperature and washed twice with deionized water.

### Preparation of paclitaxel-loaded nanoparticles by precipitation

The homogenous solutions of PVA were prepared. Specific amount of PLGA and paclitaxel was dissolved in acetone (5 ml) as the organic phase and stirred for 30 min at room temperature. Next, organic phase was added to the 20 ml of PVA solution while stirring. Afterward, organic phase was evaporated overnight and nanoparticles were collected by centrifugation at 12000 rpm for 20 min at room temperature and washed twice with deionized water.

### Characterization of nanoparticles

#### Scanning Electron Microscopy (SEM)

**Figure 1:**
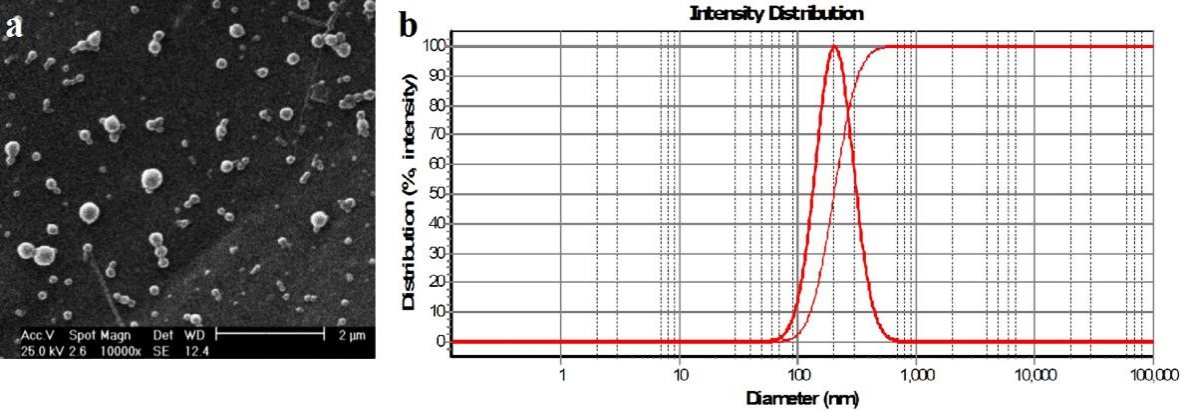
**a)**SEM image of PLGA-PTX nanoparticles **b)** DLS result of PLGA-PTX nanoparticles.

The morphology and diameter of nanoparticles were carried out using SEM as an accelerating voltage of 20.0 kv (Philips XL-30) after sputtering with gold. The diameter of nanoparticles was measured by randomly choosing 30 nanoparticles by SemAfore (4.01 demo, JEOI., Finland) software as shown in Figure 1a.

#### Dynamic light scattering (DLS)

The hydrodynamic diameter and the median nanoparticles size were obtained using DLS (Scatter Scope) as shown in Figure 1b.

## Results and Discussion

### Comparison of two methods single emulsion and precipitation

Table 1 and 2 summarizes experiments conducted in this study to investigate drug and PLGA concentrations, the amounts of PVA and solvent and sonication time which affect PLGA nanoparticles size and morphology. Emulsion solvent evaporation and nanoprecipitation/interfacial deposition are the two most common methods for the preparation of polymeric nanoparticles. These two techniques were originally developed by Vanderhoff‏ *et al*^45^ and Fessi‏ *et al*,^46^ respectively. In an study PLGA incorporated with procaine nanoparticles was prepared via nanoprecipitaion technique with the size less than 210 nm.^32^ In another study protein encapsulated in PLGA nanoparticles were prepared by phase separation method with the size around 300 nm for biomedical application.^47^ Commercial success of the various polymeric nanoformulation products developed by emulsion and nanoprecipitation were documented by Nava-Arzaluz‏ *et al*^48^ and Minost‏ *et al*.^49^ We investigated the effect of two methods of single emulsion and precipitation on nanoparticles size. The size of polymeric nanoparticles is particularly important in cancer therapy as drug delivery vehicles can enter into the tumor tissue via the EPR effect. Our results indicated that paclitaxel-loaded nanoparticles prepared with precipitation had smaller size (Table 2. No. 11) compare to single emulsion method (Table 1, No. 19). In addition, acetone is used in precipitation method which have less toxic effect than DCM applied in emulsion method.^50^ Therefore, precipitation method is suggested for medical applications.

**Table 1:** Applied parameters for preparation of paclitaxel loaded PLGA nanoparticles using single emulsion

**Number**	**PLGA** **(mg)**	**Solvent** **(ml)**	**Drug** **(mg)**	**PVA** **(W/V %)**	**Sonication time** **(min)**	**Mean Size** **(nm)**
**1**	20	3	1	1%	4	250 ± 12
**2**	20	3	1.5	1%	4	270 ± 22
**3**	20	3	3	1%	4	353 ± 24
**4**	20	3	4.5	1%	4	371 ± 39
**5**	20	3	6	1%	4	402 ± 9
**6**	60	3	3	1%	2	548 ± 10
**7**	60	3	3	1%	4	303 ± 15
**8**	60	3	3	1%	5	546 ± 17
**9**	60	3	3	1%	6	598 ± 20
**10**	10	3	1.5	1%	4	250 ± 15
**11**	30	3	1.5	1%	4	327 ± 17
**12**	40	3	1.5	1%	4	354 ± 12
**13**	50	3	1.5	1%	4	381 ± 18
**14**	60	3	1.5	1%	4	414 ± 21
**15**	30	1	1	1%	4	478 ± 10
**16**	30	2	1	1%	4	381 ± 12
**17**	30	3	1	1%	4	300 ± 18
**18**	30	4	1	1%	4	412 ± 21
**19**	30	5	1	1%	4	469 ± 14
**20**	30	6	1	1%	4	491 ± 11

### The effect of PVA

**Figure 2:**
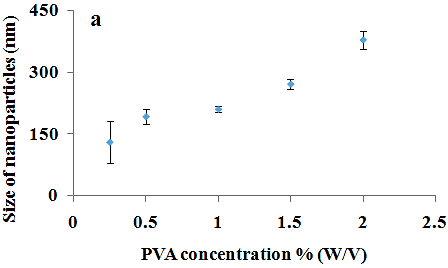
The effect of PVA concentration on nanoparticles size.

The function of PVA concentration as an effective factor on PLGA diameter is shown in Figure 2. In this experiment the applied concentrations of PVA were 0.25, 0.5, 1 and 2 W/V % whereas other parameters were constant. The nanoparticles diameter increased from about 130 nm to 378 nm (Table 2, Nos. 6-9) as the PVA concentration increased from 0.25 to 2 W/V%. It was observed that the size of the nanoparticles enhances in the precipitation method with increasing PVA concentration which can be associated with the deposition of PVA on the surface of paclitaxel-loaded nanoparticles. An increase in nanoparticle size has also been reported as the concentration of PVA enhanced.^51^

### The effect of drug concentration

**Figure 3:**
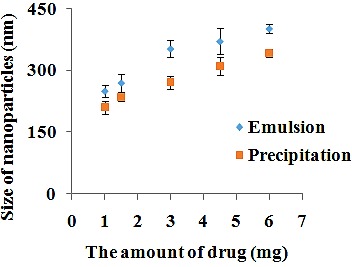
The effect of drug concentration on nanoparticles size by single emulsion and precipitation method.

The effect of the amount of drug was also examined on nanoparticles diameter. In this study the different amounts of drug were used whilst other parameters were constant by both emulsion (Table 1, Nos. 1-5) and precipitation (Table 2, Nos. 1-5) methods. As shown in Figure 3, by increasing the amount of drug from 1 to 6 mg, the nanoparticles diameter enhanced from 250 nm to 402 nm and from 210 nm to 342 nm using emulsion and precipitation methods, respectively. This increase may be because of the more content of drug available in the emulsion droplets or adsorption of drug on surface of nanoparticles in single emulsion method. In addition, increase in the amount of paclitaxel leads to larger size of nanoparticles because more solid content form after evaporation in precipitation method. The literature also confirms that increase in content of drug results in larger size of nanoparticles.^52,53^ Although some reports presented lack of relationship between size of nanoparticles and drug concentration.^50,54^

**Table 2:** Applied parameters for preparation of paclitaxel loaded PLGA nanoparticles using precipitation method

**Number**	**PLGA** **(mg)**	**Solvent** **(ml)**	**Drug** **(mg)**	**PVA** **(W/V %)**	**Mean Size** **(nm)**
**1**	20	5	1	1%	210 ± 17
**2**	20	5	1.5	1%	235 ± 10
**3**	20	5	3	1%	271 ± 16
**4**	20	5	4.5	1%	310 ± 32
**5**	20	5	6	1%	342 ± 9
**6**	20	5	1	0.25%	130 ± 70
**7**	20	5	1	0.5%	192 ± 21
**8**	20	5	1	1.5%	271 ± 16
**9**	20	5	1	2%	378 ± 29
**10**	10	5	1.5	1%	217 ± 14
**11**	30	5	1.5	1%	251 ± 12
**12**	40	5	1.5	1%	267 ± 21
**13**	50	5	1.5	1%	289 ± 18
**14**	60	5	1.5	1%	324 ± 17

### The effect of sonication time on nanoparticles size

**Figure 4:**
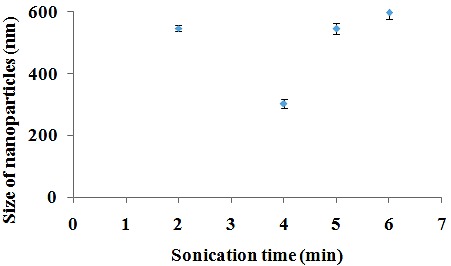
The effect of sonication time on nanoparticles size.

Polymeric nanoparticles can be synthesized via two approaches; “bottom up” or “top down”. In “bottom up”, the nanoparticles are formed from continuous deposition of molecular growth species on a nanoparticle nuclei, that is, polymer aggregation. In “Top down” synthesis, external energy sources are used to break down colloidal polymer complex structures into nanoemulsions. A commonly applied technique in the “top down” synthesis is the use of ultra-sound homogenization.^55^ In this experiment, for nanoparticles prepared by emulsion method, probe sonicator was used for 2, 4, 5 and 6 min (Table 1, Nos. 6-9) and the trend of applied sonication time on mean nanoparticles size was investigated. As shown in Figure 4, the nanoparticles size decreased from 548 to 303 nm when applied sonication time increased from 2 to 4 min. It can be attributed to the high released energy in emulsification process, resulting in the formation of smaller droplets which affect the size of polymeric nanoparticles. In addition, by increasing sonication time from 4 to 6 min, the nanoparticles size increased from 303 to 598 nm. This increase may be because of de-emulsification process^56^ or agglomeration.^57^ In a study, size of drug loaded PLGA nanoparticles was optimized by varing sonication time.^58^ Therefore, sonication time can be considered as a parameter for optimized size of nanoparticles.

### The effect of polymer concentration 

**Figure 5:**
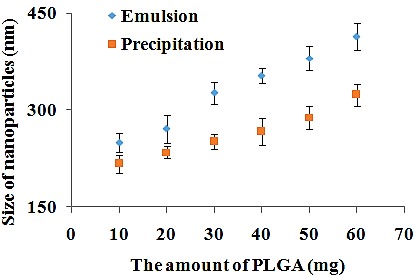
The effect of PLGA concentration on nanoparticles size prepared by single emulsion and precipitation method.

In this experiment, by increasing the PLGA concentration from 10 to 60 mg, the mean size of nanoparticles enhanced from about 250 to 414 nm (Table 1, Nos. 2, 10-14) and from about 217 to 324 nm (Table 2, Nos. 2, 10-14) in emulsion and precipitation methods, respectively (Figure 5). In both techniques, it was observed that the size of the nanoparticles has a direct relationship with the PLGA concentration. In single emulsion method, this might be attributed to the increase in viscosity of dispersed phase, resulting in a reduction of the net shear stress and prompting bigger nanodroplets. Besides, PLGA solution cannot be rapidly dispersed into the aqueous phase as the viscosity increases and result in larger nanoparticles.^59,60^ In precipitation method, frequency of collisions increase which leads to fusion of nanoparticles as concentration of polymer enhances.^61^

### The effect of organic to aqueous phase volume ratio

**Figure 6:**
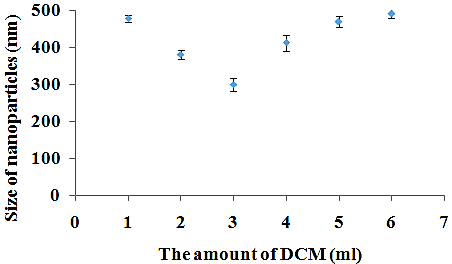
The effect of aqueous to organic phase volume ratio on nanoparticles size.

In our experiments, the nanoparticles size demonstrated an initial decrease from 478 nm to 300 nm as the amount of the organic phase enhanced from 1 to 3 ml (as shown in Figure 6 and Table 1, Nos. 15-17). The reason why the nanoparticles size decreased from 478 nm to 300 may be attributed to viscosity of organic phase. Increasing organic phase results in decrease in concentration of polymer and consequently decreases in size. However, by further increasing the amount of the organic phase from 3 to 6 ml, the particle size enhanced from 300 nm to 491 nm (Table 1, Nos. 17-20). This may be because of an enhanced time for the evaporation of the organic phase. In other words, a higher amount of solvent may cause ostwald ripening of the nanoemulsions before solvent evaporation of organic phase.

## Conclusion

This study compared two methods for preparation of paclitaxel loaded PLGA nanoparticles including single emulsion and precipitation. The results indicated that precipitation method results in smaller nanoparticles compared to emulsion one. In addition, the effect of the various parameters on the size of the nanoparticles was investigated The results demonstrated that the concentration of the PLGA polymer, drug and PVA had a direct relationship with the size of nanoparticles whereas sonication time and organic phase to aqueous volume ratio needed to the optimization to obtain the nanoparticles with smaller sizes.

## Acknowledgments

This work was supported by Tehran University of Medical Sciences, Grant No. 96-01-87-34138.

## Ethical Issues

Not applicable.

## Conflict of Interest

The authors declare no conflict of interests.
